# Relationship Between Age at First Calving and 305-Day Milk Yield in Hungarian Holstein-Friesian Cows: Trends and Genetic Parameters

**DOI:** 10.3390/ani15243648

**Published:** 2025-12-18

**Authors:** Szabolcs Albin Bene, Zsolt Jenő Kőrösi, László Bognár, József Péter Polgár, Ferenc Szabó

**Affiliations:** 1 Institute of Animal Sciences, Georgikon Campus, Hungarian University of Agriculture and Life Sciences, Deák Ferenc u. 16, H-8360 Keszthely, Hungary; 2 National Association of Hungarian Holstein Friesian Breeders, Lőportár u. 16, H-1134 Budapest, Hungary; 3 Department of Animal Sciences, Albert Kázmér Faculty of Agriculture and Food Sciences, Széchenyi István University, Vár t. 2, H-9200 Mosonmagyaróvár, Hungary

**Keywords:** Holstein-Friesian, age at first calving, 305-day milk yield, heritability, breeding value, phenotypic and genetic trend

## Abstract

Age at first calving (AFC) and 305-day milk yield in the first lactation (MY) data of 18,545 Holstein-Friesian cows born between 2008 and 2018 in six herds were evaluated. The effects of some genetic and environmental factors, population genetic parameters, breeding value (BV), and phenotypic and genetic trends of AFC and MY traits were estimated. The GLM method (ANOVA Type III) and BLUP animal model were used for the estimations. One-way linear regression analysis was used for trend calculations. The adjusted overall mean value (±SE) of the AFC and MY traits was 25.19 ± 0.02 months and 10,287.14 ± 24.79 kg, respectively. The percentage proportion contribution of the different factors in the phenotype in the case of AFC was as follows: herd 94.41%, birth year of cow 3.26%, birth season of cow 1.39%, and sire 0.71%. For MY, the contribution was as follows: herd 89.17%, birth season of cow 5.38%, birth year of cow 4.09%, and sire 1.05%. The heritability of AFC and MY traits by two different models proved to be moderate (0.26 ± 0.02, 0.19 ± 0.01 and 0.30 ± 0.02, 0.34 ± 0.01, respectively). There were relatively small differences between the sires in the estimated BV for the traits AFC and MY. The phenotypic and genetic correlations between AFC and MY traits were weak (between −0.05 and −0.16). Based on the phenotypic trend calculation, AFC showed a decreasing direction (−0.12 months per year) and MY an increasing direction (+42.30 kg per year). However, the genetic trend was very slightly decreasing for AFC (−0.00 and −0.05 months per year) and slightly increasing for MY (+5.52 and +16.49 kg per year) over the period studied.

## Simple Summary

The subject of this manuscript is the age at first calving in Holstein-Friesian cows, which is an important trait from an economic point of view. It is true that if a cow calves at a younger age, she will produce earlier and the costs of rearing her will be refunded earlier. This paper shows and discusses the environmental and genetic factors influencing age at first calving, their rates, the trend, and the relationship with 305-day milk yield of this trait. Our findings confirm a slight, but consistent, phenotypic decrease in age at first calving and increase in milk yield among Hungarian Holstein-Friesian cows. Despite moderate heritability, significant environmental effects, particularly herd management, largely influenced these traits. These results suggest that while age at first calving may not yet be a direct selection criterion, it holds promise as a complementary trait for improving herd productivity and efficiency.

## 1. Introduction

The Holstein-Friesian (HF) is large-bodied, medium mature cattle breed [1]. It was bred in the USA in the 1800s and is undoubtedly the most recognized and widespread dairy breed in the world. In breeding practice we see that heifers of this breed are increasingly being bred at younger ages, with the age at first calving (AFC) decreasing each year [2]. AFC is a defining moment that describes an important event in the cow’s life, as it marks the beginning of the dairy cow’s productive career [3].

There are many previous studies in the literature that have evaluated the effect of different factors on AFC: live weight [4], herd [5], parity of dam [6], etc. The sire can also influence the AFC of his daughters [7]. In the study by Santos et al. [8], the genotype × environment interaction had a significant effect on AFC.

AFC is associated with many traits. Kirkpatrick and Berry [9] found a weak phenotypic correlation (r_p_ = 0.14) between the twinning and AFC. According to van der Heide et al. [10], the genetic correlation (r_g_) between longevity and AFC was 0.26. AFC showed a close r_g_ with some conformation and udder traits [11,12], longevity [13], growth traits [14], calving difficulty [15], calving interval and conception [16,17], and milk protein polymorphism [18].

According to many literature sources, AFC is related to milk production (MP). In the study by Easthem et al. [19] and Pirlo et al. [20], an increase in AFC was associated with an increase in MP, i.e., there was a direct proportionality between the two traits. Similarly, Van Eetvelde et al. [21] described a positive association between AFC and MP traits, with a plateau between 34 and 42 months. In contrast, in the study by Krpalková et al. [16], the earliest-calving cows (AFC = 735 days) had the highest milk production (8126 kg). Cows that calved later (AFC = 825 days) had a lower milk yield (7327 kg). According to Hutchinson et al. [22], the r_g_ between the traits AFC and MP in HF cows was −0.43. In the study by Ruiz-Sanchez et al. [23] the r_p_ and r_g_ between the AFC and MP traits were −0.11 and −0.44, respectively. Several other studies reported an optimum for MP at an AFC of 22 to 26 months, with similar or even lower MPs in older cows [24,25].

There are many different values of heritability (h^2^) reported in the literature for AFC in different breeds [26]. According to most literature sources [27,28], the h^2^ of AFC is quite low or moderate. Similar to the previous trait, the h^2^ of the MP trait is also moderate in the literature sources [29,30,31,32]. In addition, the h^2^ data of the AFC and MP traits are variable in the literature.

As AFC is largely a breeder decision, there is very little information in the international literature on the breeding value (BV) of bulls with the AFC trait [33,34]. Bormann and Wilson [35] published BV’s of sires for AFC, ranging from −46.6 to +45.9 days. BVs for the trait MP are usually found in INTERBULL [36] publications, and their numerical data are found in very few of the literature sources.

When analyzing the relevant literature, it seems that AFC has shown a decreasing and MP an increasing trend in all dairy cattle breeds in the recent period [37,38]. In contrast, the relationship between r_p_ and r_g_ is not clear. In our opinion, these require clarification using a large database and complex mathematical procedures.

The MP of HF cows is well known and has been reported by many previous sources [1,9,12,20,23,26]. Therefore, the results of the relevant literature sources will not be detailed here.

Therefore, the aim of this study was to examine the AFC and 305-day milk yield in the first lactation (MY) data of Hungarian HF cows on a large national database. During the work, six questions were formulated, which we wanted to answer based on the results:

1. What influence do sire, herd, year of birth, and season have on the AFC and MY traits of HF cows?
2. What is the h^2^ of the AFC and MY traits?
3. What are the differences in BV for the AFC and MY traits between HF sires?
4. Are there any differences in the ranking of the sires due to the different BV models?
5. What is the phenotypic and genetic relationship between the AFC and MY traits?
6. What are the trends (phenotypic and genetic) for the AFC and MY traits in the tested Hungarian HF population?

## 2. Materials and Methods

### 2.1. The Processed Data and the Examined Traits

The data sources for this study were six large-scale HF herds provided by the National Association of Hungarian Holstein Friesian Breeders (NAHHFB) in Hungary. Data from 18,545 HF cows born between 2008 and 2018 were included in the study. The cows were the offspring of 732 sires and 13,819 dams (Table 1).

The AFC of cows was calculated as the distance between the date of birth and the date of first calving [33,34]. Similar to the method used by Hutchinson et al. [22], Hossein-Zadeh [38], and Ferrari et al. [26], only data from cows aged 18 to 40 months at first calving were used.

The 305-day milk yield in the first lactation (MY) data were obtained from the national database of the NAHHFB [36]. Only data from cows whose milk yield in the first lactation was between 5000 and 18,000 kg were used.

### 2.2. Farm Management and Housing Technology

The Holstein cows examined were kept in a free-range housing facility, group-based according to their lactation status and production, and milked in a milking parlor. The feedings were based on a TMR (Total Mixed Ration), which consisted of corn silage, alfalfa or ryegrass hay, and concentrates.

### 2.3. Descriptive Statistic

Descriptive statistics involved simple mathematical methods using the SPSS 27.0 [39] software (Descriptive statistic module).

The Kolmogorov–Smirnov test was used to check the normality and the Levene test to check the homogeny of variance of the data for the AFC and MY traits.

### 2.4. The Used Models

During the study, two models were used to estimate variance components, population genetic parameters, and BVs. One was a GLM model and the other was a BLUP animal model [40]. The used models are presented in Table 2.

### 2.5. Estimation with GLM Method

#### 2.5.1. Examining the Effects of Different Factors

The effects of the different genetic and environmental factors on the AFC and MY traits were evaluated by univariate analysis of variance (GLM, general linear model; hereafter the GLM method). The random effect was the sire, while the other studied factors—herd, birth year of cow, and birth season of cow—were considered as fixed effects [41]. The GLM model used was as follows (Equation (1)):

(1) $${\hat{y}}_{hijk}=\mu +F_{h}+Y_{i}+M_{j}+S_{k}+e_{hijk}$$

where ŷ_hijk_ = the estimated AFC or MY of the cow born in herd “h”, year “i”, and season “j” of sire “k”; μ = mean of AFC or MY trait; F_h_ = herd effect (fixed); Y_i_ = birth year of cow effect (fixed); M_j_ = birth season of cow effect (4 seasons, namely, winter = December + January + February; spring = March + April + May; summer = June + July + August; Autumn = September + October + November) (fixed); S_k_ = sire effect (random); e_hijk_ = random error.

The study examined the effect of the genetic and environmental factors on the named traits. In cases where the GLM showed a significant difference, the Tukey test was used to detect differences between groups in the case of homogeneous variance, and the Tamhene test was used in the case of non-homogeneous variance.

The Microsoft Excel 2007 and Word 2007 software packages were used to prepare the database. The SPSS 27.0 [39] statistical software package was used to evaluate the effect of different factors.

#### 2.5.2. Population Genetic Parameter Estimation with GLM Method

The previous GLM model was used to estimate the three variance components, where the genetic variance (σ^2^_d_), the environmental variance (σ^2^_e_), and the phenotypic variance (σ^2^_p_) were estimated. For the estimations, the ANOVA Type III procedure was used. This procedure is mathematically equivalent to Harvey’s sire model [42].

The genetic variance (σ^2^_d_) was calculated as follows (Equation (2)):

(2) $${\sigma^{2}}_{d}=\left[\frac{MS_{sire}-MS_{e}}{k_{1}}\right]\times 4$$

where σ^2^_d_ = genetic variance; MS_sire_ = the mean square value of sire from the ANOVA table; MS_e_ = the mean square value of the residual (error) from the ANOVA table, i.e., MS_e_ = σ^2^_e_; the k_1_ coefficient was calculated from the number of animals and the sire’s degree of freedom [42].

From the variance data obtained, h^2^ was calculated using the following method (Equation (3)):

(3) $$h^{2}=\frac{{\sigma^{2}}_{d}}{{\sigma^{2}}_{d}+{\sigma^{2}}_{e}}=\frac{{\sigma^{2}}_{d}}{{\sigma^{2}}_{p}}$$

The GLM method and calculation procedure used were the same as described in detailed in our previous paper [43]. In the case of the GLM method, the SPSS 27.0 [39] software was used for the estimates.

#### 2.5.3. Breeding Value Estimation with GLM Method

Based on the guidelines of Tőzsér and Komlósi [44], the estimated value of BV with the GLM method (BV_GLM_) was calculated using the following formula (Equation (4)):

(4) $$BV_{GLM}=\frac{2\times n\times h^{2}}{4+(n-1)\times h^{2}}\times ({\bar{X}}_{i}-\bar{X})$$

where BV_GLM_ = breeding value of sire in AFC or MY trait, estimated with the GLM method; n = number of progeny of the sire; h^2^ = heritability; X_i_ = the average AFC or MY of the sire’s offspring group; X = mean value of the AFC or MY of the contemporary offspring population.

Based on the instructions of Tőzsér and Komlósi [44], the reliability (b) of the estimated BV’s was calculated as the follows (Equation (5)):

(5) $$b=\sqrt{\frac{n\times h^{2}}{4+(n-1)\times h^{2}}}$$

where b = reliability of the estimated BV; n = number of progeny of the sire; h^2^ = heritability.

For reasons of size, the BV’s are only shown for the 10 sires with the most progeny.

### 2.6. Estimation with the BLUP Animal Model

#### 2.6.1. Population Genetic Parameters Estimation with the REML Method

The population genetic parameters were analyzed using the maximum likelihood (REML) approach with the DFREML 3.0 [45] software.

Similar to Djedovic et al. [46], the effect of fixed factors (as in the GLM method) on the examined traits was tested using the step-by-step method, so that the models used in this research only included factors that showed a statistically significant effect within the mentioned procedure. The effect of the individual (animal effect, cow) was included as a random factor.

Based on the above, the following model was used to estimate the variance components (Equation (6)):

(6) $${\hat{y}}_{hijo}=\mu +F_{h}+Y_{i}+M_{j}+a_{o}+e_{hijo}$$

where ŷ_hijo_ = the phenotypic expression of the AFC or MY trait; μ = mean of the AFC or MY trait; F_h_ = herd effect (fixed); Y_i_ = birth year of cow effect (fixed); M_j_ = birth season of cow effect (fixed) (as in Equation 1); a_o_ = animal (cow) effect (random); e_hijo_ = random error.

The σ^2^_d_, σ^2^_e_, and σ^2^_p_ values were determined during the estimation using the REML method. Similar to the GLM method, the h^2^ value was calculated with Equation (3).

#### 2.6.2. Breeding Value Estimation with BLUP Animal Model

The BLUP animal model (BV_BLUP_) was used for estimation of BVe of the studied traits. Two matrices were created; the former included pedigree data for sires, dams, grandparents, full sibs, and half sibs. The database matrix included the same fixed effects as in the GLM method (as above) and the AFC and MY data. The random effect was the individual (cow) [47]. The general formula of the used BLUP animal model was as follows (Equation (7)):

(7) y=Xb+Zu+e

where y = vector of observation; b = vector of fixed effects; u = vector of random effects; e = error vector; X = matrix of fixed effects; Z = matrix of random effects.

Based on the guidelines of Szőke and Komlósi [48], the MTDFREML software [49] was used to run the BLUP animal model for BV estimation. The MTDFREML software used automatically determined the BVs, which were copied from the result files for further calculations.

Similar to the GLM method, the reliability (b) of the estimated BV_BLUP_ was estimated using the formula shown in Equation (5).

Also, similar to the GLM method, for reasons of size, the BV_BLUP_ values are only shown for the 10 sires with the most progeny.

### 2.7. Comparison the Ranking of Sires

Using the GLM method and the BLUP animal model two–two different rankings were obtained based on the estimated BV of the sires in the traits AFC and MY. In line with the studies of Núnez-Dominguez et al. [50], the effect of the model on sire rank was calculated with rank correlation method [51].

### 2.8. Phenotypic and Genetic Correlations

The phenotypic correlation coefficient (r_p_) was based on measured data. The genetic correlation coefficient (r_g_) was based on the BV’s of the AFC and MY traits. The correlation coefficients were calculated according to the instructions of Tőzsér and Komlósi [44].

The SPSS 27.0 [39] software was used for correlation calculations.

### 2.9. Phenotypic Trend Estimation

To calculate the phenotypic trend, the AFC and MY data of cows born in the same year were averaged. The annual means of AFC and MY (dependent variable, Y) were plotted in the coordinate system. The X value (independent variable) was the birth year of cows. One-way linear regression analysis was used to find the best-fitting line to the points. The constant (a), slope (b), and goodness of fit (R^2^) values and their standard error (SE) and statistical reliability were also estimated.

### 2.10. Genetic Trend Estimation

Two different methods were used to calculate the genetic trends. On the one hand, the BV of the sires in AFC and MY traits born in the same year and, on the other hand, the BV of the entire population in AFC and MY traits born in the same year were averaged and plotted on a coordinate system (dependent variable, Y). The independent variable (X) was the birth year of sires or birth year of the entire population.

For sires, BV (and genetic trend) was calculated separately using the GLM method and using the BLUP animal model.

Similar to the phenotypic trend estimation, a one-way linear regression analysis was used to fit a line to the resulting points. According to Ostler et al. [52], the constant value (a), the slope value (b), and the goodness of fit value (R^2^) were determined as well as their statistical reliability.

Estimates of genetic trends were made for the period 2001–2016 for sires and 2008–2018 for the entire population.

Both phenotypic and genetic trends of the AFC and MY traits were calculated using the SPSS 27.0 [39] software.

## 3. Results and Discussion

### 3.1. Effect of Different Factors

As can be seen from the data in Table 3, the arithmetic mean (±SE) of AFC of the HF cows was 25.0 ± 0.0 months. Subtracting the average gestation length of the cows (285 days or 9.5 months) from the AFC, it can be concluded that the HF heifers in Hungary were exposed to breeding at an average age of 15.5 months. This result is similar to that found by Berry and Cromie [15] but lower than that published by Ferrari et al. [26]. Compared to the data from previous works, the AFC (month) of the HF cows in the different sources was as follows: 22.6 [53], 23.5 [54], 25.6 [28], 26.0 [27], 26.1 [20], 26.5–30.0 [37], and 26.9 [55].

The mean of MY was 10,179.4 ± 15.1 kg. Most of the relevant literature sources [29,47,56] reported similarly high MY values.

In all cases, a significant effect (*p* < 0.01) was found between the examined random and fixed factors on the AFC and MY traits (Table 4). The percentages of observed effects in phenotype were as follows: herd (AFC 94.41%, MY 89.17%), birth year of cow (AFC 3.26%, MY 4.09%), birth season of cow (AFC 1.39%, MY 5.38%), and sire (AFC 0.71%, MY 1.05%). However, compared to the data reported by Mohd Nor et al. [7], the magnitude of the sire effect is smaller in our work.

Table 5 summarizes the effect of environmental factors on the AFC and MY traits. The adjusted mean value (±SE) of the AFC and MY traits obtained by the GLM method was found to be 25.2 ± 0.0 months and 10,287.1 ± 24.8 kg, respectively.

Of the studied herds, herd number 5 had the highest AFC (27.5 ± 0.1 months), which was 2.5–3.3 months higher than that observed in the other herds. In the case of MY, the difference between the best herd (herd number 1, 11,539.3 ± 70.9 kg) and the worst herd (herd number 5, 8417.9 ± 58.7 kg) was 3122 kg. The results for the influence of herd on AFC were similar to those published by Dobos et al. [4] and Ettema and Santos [5].

The AFC of cows born in the early years (year 2008–2009) were 1.1 months higher than that of cows born in the later years (year 2017–2018).

Regarding the birth year of cows, the MY trait of the cows was quite balanced (except for year 2008, where the smallest MY was calculated). The largest MY (10,963.6 ± 103.3 kg) was found in year 2018. The annual MY results were similar to the data reported by Ansari-Lari et al. [37] and Hossein-Zadeh [38].

### 3.2. Breeding Values and Heritabilities of the Examined Traits

Table 6 shows the means of the AFC and MY traits of the sire’s progeny groups. The two different models gave different results between the estimated BV of the sires.

Estimation by the GLM method showed the lowest AFC (24.7 ± 0.2 months) in the progeny group of the sire with registration number 25863. The highest AFC (25.8 ± 0.2 months) was found in the progeny group of the sire with registration number 21556. The difference between the two extremes was 1.1 months, which is much less than previously observed [30].

According to the previous results, in case of the AFC trait, a difference of 2.0 and 1.2 months was found between the two BV extremes (by sires with registration numbers 25863 and 21556) using the GLM method and the BLUP animal model, respectively. The BV values of AFC estimated with the BLUP animal model were lower than the data published by Bormann and Wilson [35] in Angus and by Bognár et al. [47] in HF cows.

In the case of MY, the lowest production (9226.1 ± 165.7 kg) was shown by daughters of the sire with registration number 21556. It was shown in the previous sections that daughters of this sire were the earliest to be taken into breeding. The highest MY (11,189 ± 150.1 kg) was obtained in the progeny group of sire 27494. The difference between the highest and lowest MY of the progeny groups was almost 2000 kg.

Regarding the BVs estimated with both the GLM method and the BLUP animal model, the highest values (+1636.8 kg and +961.5 kg, respectively) were estimated for the sire with registration number 27494 in the case of the MY trait. Quite large differences (about 3500 kg and 2400 kg) were found between the BVs of the sires estimated with the two different models.

Subsequently, a strong and positive rank correlation (in AFC and MY, r_rank_ = 0.91 and 0.87, respectively) was found between the sire rankings obtained in the two different models.

In the studied HF population, the estimated h^2^ values (±SE) of the AFC and MY traits were moderate (Table 7).

In the case of AFC, the h^2^ value estimated with the GLM method was 0.26 ± 0.02, while that estimated with the BLUP animal model was 0.19 ± 0.01. In contrast to our results, the h^2^ of the AFC trait is usually low in the relevant literature sources: HF 0.10, 0.03 [27,28], Brown Swiss 0.08 [57], and Ayrshire 0.09 [58]. In summary, the h^2^ estimates for AFC in our study were slightly higher than some published in the literature [27,28,55].

Although a relatively small difference was found between the h^2^ values estimated with the two models, the h^2^ estimates differed significantly between the GLM and BLUP models. The reason for this may be that the GLM method estimated the genetic variance based on the sire data, whereas the BLUP model estimated the genetic variance based on total population data.

In the case of MY, slightly higher h^2^ values (0.30 ± 0.02 and 0.34 ± 0.01) were estimated than previously. Most of the relevant literature sources reported similar h^2^ values for the MY trait. These estimates are higher than those published by Dematawewa and Berger [32], who reported an h^2^ value of 0.20 for MY. They also exceed the results of Roman et al. [31] and Abdallah et al. [59], who reported h^2^ estimates ranging from 0.17 to 0.37 for MP traits. Erfani-Asl et al. [60] reported even smaller h^2^ values (0.13–0.16) for MP traits than previous results.

As the h^2^ of AFC is slightly lower than that of MY but greater than zero (0.19–0.26), it also offers a selection opportunity for this trait. However, based on the data, we would expect genetic progress to be slower than what is achievable in terms of increasing milk yield.

### 3.3. Phenotypic and Genetic Correlations

The r_p_ and r_g_ values between the AFC and MY traits in the studied HF population are shown in Table 8. According to the results, there was a weak but significant between r_p_ and r_g_ (from −0.05 to −0.16) and the AFC and MY traits. In our study, the negative low r_g_ between the AFC and MY traits indicates a weak association.

Our results are similar to data of Hutchinson et al. [22] and Ruiz-Sanchez et al. [23], who found −0.43 and −0.44 values of r_g_ between the AFC and MY traits. Furthermore, our results showed similarities with the studies of Krpalková et al. [16] and Curran et al. [24], who found the highest milk production in cows that were introduced into breeding earliest. In contrast, our results differ from those of Pirlo et al. [20], Eastham et al. [19], and Van Eatvelde et al. [21], who found a positive relationship between AFC and MY.

The correlation coefficients were statistically proven to be small and negative. However, the negative signs indicate that earlier exposure to breeding may increase milk production in the first lactation.

### 3.4. Phenotypic and Genetic Trends

**Figure 1:**
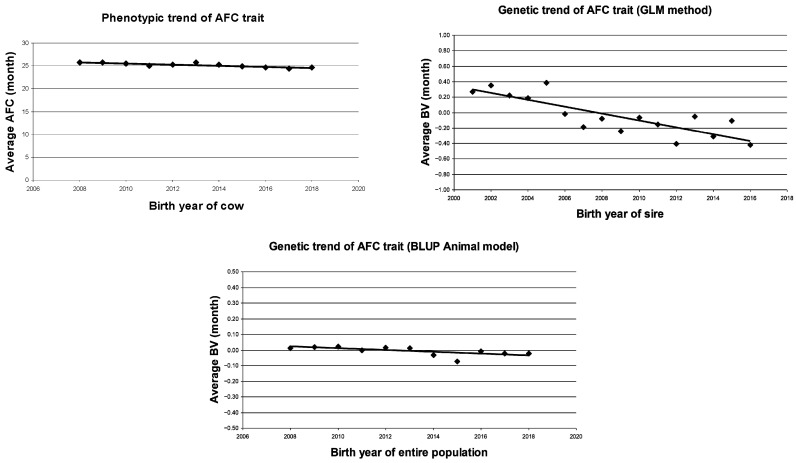
Phenotypic and genetic trends in age at first calving trait in Holstein-Friesian cows. AFC = average age at first calving (month); BV = breeding value.

**Figure 2:**
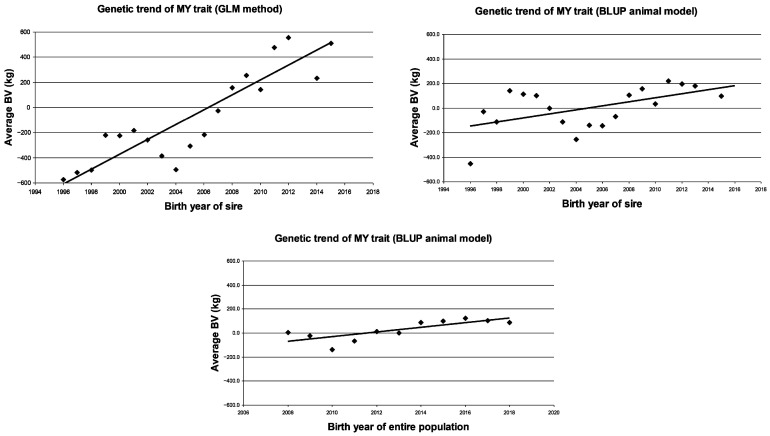
Phenotypic and genetic trends in 305-day milk yield in the first lactation trait in Holstein-Friesian cows. MY = 305-day milk yield in the first lactation (kg); BV = breeding value.

The results of the phenotypic trend calculation in the studied HF population clearly showed a decreasing trend in the AFC trait and an increasing trend in the MY trait (Table 9 and Figure 1 and Figure 2).

Taking into account the phenotypic trends, AFC decreased by −0.12 months per year (b = −0.12 ± 0.02; *p* < 0.01). It seems that MY was increasing by +42.3 kg per year (b = +42.3 ± 24.8; NS), but this trend was not statistically proven. The fitting of the phenotypic trend was strong and significant (R^2^ = 0.75; *p* < 0.01) in the case of the AFC, but it was not significant in the case of MY (R^2^ = 0.25; NS).

Similarly to the previous result, the genetic trend (based on BV of sires) showed a yearly decrease in the AFC trait and a yearly increase in the MY trait. There was an average decrease of −0.05 months per year (b = −0.05 ± 0.01; *p* < 0.05) and +59.1 kg (b = +59.1 ± 6.7; *p* < 0.01), respectively, as estimated by the GLM method. The fitting of these trends of the AFC and MY traits showed a medium or strong (R^2^ = 0.69 and 0.81) and significant value (*p* < 0.01).

However, when the BLUP animal model was used, the trend estimate based on the sire’s BV of the AFC trait did not result in a significant change, i.e., the slope was almost zero and the fit of the equation was very poor (R^2^ = 0.12; NS). In contrast, the genetic trend based on the sire’s BV of the MY trait was increasing (b = +16.5 ± 6.2; *p* < 0.05) and had a significant fit (R^2^ = 0.29; *p* < 0.05).

A partially similar result was obtained for the BV of all animals, where a very low value and a negative genetic trend was found in AFC (b = −0.01 ± 0.00; *p* < 0.01) and MY (b = +19.10 ± 5.21; *p* < 0.01).

Based on these results, it appears that there were very small genetic changes in the AFC of the HF cows during the evaluated period.

Compared to the literature data, the study by Ansari-Lari et al. [37] showed a significant decrease in the AFC of HF cows in Iran from 30 months (in year 2000) to 26 months (in the year 2005). Hare et al. [3] found a decrease in the AFC of the HF, Brown Swiss, and Jersey breeds in the USA in the period 1980–2004. Hossein-Zadeh [38] reported a decreasing phenotypic (−0.08 month/year) and genetic (−0.01 month/year) trend for AFC in the HF breed between 1990 and 2007. Amimo et al. [58] reported a decreasing genetic trend (−0.01 month/year) in Ayrshire cows in Kenya.

Our results are confirmed, as a similar trend has been published for dairy herds by Hare et al. [3], Ansari-Lari et al. [37], Hossein-Zadeh [38], and Ostler et al. [52].

## 4. Conclusions

In our study we wanted to see how simultaneous efforts by breeders to increase milk production and reduce AFC would lead to changes in the latter trait in the HF breed.

Based on the results of this study, AFC did not decrease genetically to a significant extent. This may be due to the low h^2^ estimates, the low R^2^ genetic trends, and the weak correlation with milk production. Other possible factors include the bias introduced by the genotype × environment effects, which imply limited selection pressure on AFC.

Taken together, this may have resulted in only a small phenotypic improvement in AFC compared to the increase in milk production.

As AFC is an important trait from many perspectives (e.g., economic, reproductive biological, etc.), it would be useful to investigate the feasibility of further reducing AFC and its economic and biological limitations.

Our findings confirm a very slight, but consistent, phenotypic decrease in AFC and an increase in MY among Hungarian HF cows. As a consequence of moderate heritability, significant environmental effects, particularly herd management, influenced these traits to a large extent. These results suggest that although AFC may not yet be a direct selection criterion, it has the potential to complement other traits in improving herd productivity and efficiency.

When we compare our results with those in the literature, we find that some are consistent with certain studies while others are not. The possible reasons for the differences may be due to differences in the population and management.

Overall, the results of our study show that, despite a slight phenotypic decrease, the AFC of Holstein cows did not change significantly genetically over time during periods of increased milk production. In other words, the animals did not calve at a notably younger age due to their genotype. Given the significant economic interest in improving precocity, revising current selection practices from this perspective poses a great challenge for dairy industry.

## Figures and Tables

**Table 1:** Structure of the initial Holstein-Friesian population database.

Factors Examined	Used Database
Test period by date of birth of cows	2008–2018
Number of herds	6
Number of cows	18,545
Number of sires (sire of cow)	732
Date of birth of sires	2001–2016
Average number of cow progeny per sire	25.3
Number of dams (dam of cow)	13,819

**Table 2:** The models used for the estimations.

Components in the Model	GLM Method	BLUP Animal Model
Random effects		
- sire (sire of cow)	+	-
- cow (animal)	-	+
Fixed effects		
- herd	+	+
- birth year of cow	+	+
- birth season of cow	+	+
Pedigree matrix		
- sire (sire of cow)	-	+
- dam (dam of cow)	-	+
- full sibs, half sibs	-	+
- grandparents	-	+
Examined traits		
- AFC	+	+
- MY	+	+

+ = the model includes this effect; - = the model does not include this effect; AFC = age at first calving; MY = 305-day milk yield in the first lactation.

**Table 3:** Statistical characteristics of the examined traits of Holstein-Friesian cows.

Parameters	AFC (Month)	MY (kg)
N	18,545	18,545
Mean	25.00	10,179.36
Standard error (SE)	0.02	15.14
Standard deviation (SD)	2.32	1856.57
Coefficient of variation (cv%)	9.28	18.23
Median	24.57	10,216.02
Minimum	18	5000.0
Maximum	40	18,000.0
Levene test (*p*) #	0.00	0.06
Kolmogorov–Smirnov test * (*p*)	0.20	0.11

# If *p* > 0.05, homogeneity is confirmed; If * *p* > 0.05, a normal distribution is confirmed; AFC = age at first calving; MY = 305-day milk yield in the first lactation.

**Table 4:** Influence of different factors on the examined traits of Holstein-Friesian cows.

Trait	Classes	AFC		MY	
Factor		The Effect and Rate of Different Factors in the Phenotype			
		*p*	%	*p*	%
Sire of cow	732	<0.01	0.71	<0.01	1.05
Herd	6	<0.01	94.41	<0.01	89.17
Birth year of cow	11	<0.01	3.26	<0.01	4.09
Birth season of cow	4	<0.01	1.39	<0.01	5.38
Residual	-	-	0.23	-	0.31
Total	-	-	100.00	-	100.00

AFC = age at first calving; MY = 305-day milk yield in the first lactation.

**Table 5:** Environmental effects on the examined traits of Holstein-Friesian cows.

**Trait**	**N**	**AFC (Month)**		**MY (kg)**	
Adjusted overall mean (±SE)	18,545	25.19 ± 0.02		10,287.14 ± 24.79	
**Environmental Factors**		**Mean (±SE)**	**DOM**	**Mean (±SE)**	**DOM**
Herd (code)					
- 1	1630	^a^ 24.81 ± 0.08	−0.36	^a^ 11,539.29 ± 70.91	+1252.15
- 2	6056	^b^ 24.17 ± 0.04	−1.00	^b^ 10,102.33 ± 38.30	−184.81
- 3	1992	^c^ 25.01 ± 0.07	−0.16	^b^ 10,385.90 ± 62.19	+98.76
- 4	4214	^d^ 24.93 ± 0.05	−0.24	^c^ 10,073.22 ± 46.49	−213.92
- 5	3303	^e^ 27.48 ± 0.06	+2.31	^d^ 8417.94 ± 58.72	−1869.20
- 6	1350	^c^ 24.62 ± 0.08	−0.55	^e^ 11,203.78 ± 68.29	+916.64
Birth year of cow					
- 2008	1110	^a^ 25.72 ± 0.13	+0.55	^a^ 9954.10 ± 122.31	−333.04
- 2009	1369	^b^ 25.70 ± 0.11	+0.53	^b^ 10,333.41 ± 110.32	+46.27
- 2010	1469	^b^ 25.53 ± 0.10	+0.36	^c^ 10,129.88 ± 97.00	−157.26
- 2011	1463	^c^ 25.07 ± 0.09	−0.10	^a^ 10,142.34 ± 84.59	−144.80
- 2012	1695	^d^ 25.27 ± 0.08	+0.10	^de^ 10,550.30 ± 76.08	+263.16
- 2013	1661	^b^ 25.74 ± 0.08	+0.57	^d^ 10,314.06 ± 73.41	+26.92
- 2014	1810	^c^ 25.21 ± 0.07	+0.04	^de^ 10,241.00 ± 66.40	−46.14
- 2015	1829	^d^ 24.93 ± 0.08	−0.24	^d^ 10,069.09 ± 69.18	−218.05
- 2016	1896	^e^ 24.66 ± 0.09	−0.51	^e^ 10,054.73 ± 77.14	−232.41
- 2017	2104	^f^ 24.45 ± 0.10	−0.72	^f^ 10,405.11 ± 87.13	+117.97
- 2018	2139	^e^ 24.60 ± 0.12	−0.57	^g^ 10,963.64 ± 103.28	+676.50
Birth season of cow					
- winter	4773	^a^ 25.26 ± 0.04	+0.08	^a^ 10,322.21 ± 35.59	+35.07
- spring	3272	^a^ 25.16 ± 0.04	−0.01	^b^ 10,154.08 ± 39.18	−133.06
- summer	4926	^a^ 25.19 ± 0.04	+0.01	^b^ 10,233.81 ± 33.63	−53.33
- autumn	5574	^b^ 25.08 ± 0.03	−0.09	^c^ 10,438.12 ± 32.00	+150.98

Values that do not contain the same letter are significantly (*p* < 0.05) different from each other; AFC = age at first calving; MY = 305-day milk yield in the first lactation; DOM = deviation from overall mean.

**Table 6:** Sire effect on the examined traits of Holstein-Friesian cows.

Trait	Sire of Cow (Reg. Number)	N *	GLM Method			BLUP Animal Model	
			Mean of Progeny Group (±SE)	BV_GLM_	b	BV_BLUP_	b
AFC (month)	- 16530	117	25.7 ± 0.2	+0.8	0.94	+0.3	0.94
	- 19324	121	25.1 ± 0.2	−0.2	0.95	−0.5	0.95
	- 19530	281	25.2 ± 0.1	+0.1	0.98	+0.2	0.98
	- 21556	119	25.8 ± 0.2	+1.1	0.94	+0.7	0.94
	- 21727	127	25.2 ± 0.2	+0.0	0.95	−0.3	0.95
	- 22084	174	25.4 ± 0.1	+0.4	0.96	+0.5	0.96
	- 23288	135	25.1 ± 0.2	−0.2	0.95	−0.2	0.95
	- 25863	121	24.7 ± 0.2	−0.9	0.95	−0.5	0.95
	- 27494	163	25.2 ± 0.2	−0.1	0.96	+0.4	0.96
	- 28388	143	25.3 ± 0.2	+0.2	0.95	+0.6	0.95
	- AOM ± SE	18,545	25.2 ± 0.0	-	-	-	-
Rank-correlation value (r_rank_)				0.91 (*p* < 0.01)			
MY (kg)	- 16530	117	9267.3 ± 190.4	−1703.7	0.94	−1114.3	0.94
	- 19324	121	10,403.5 ± 172.9	+204.8	0.95	+778.4	0.95
	- 19530	281	10,667.9 ± 108.7	+714.9	0.98	+894.5	0.98
	- 21556	119	9226.1 ± 165.7	−1872.6	0.94	−1443.0	0.94
	- 21727	127	9784.8 ± 158.6	−887.3	0.95	−448.0	0.95
	- 22084	174	10,388 ± 130.9	+183.9	0.96	+379.8	0.96
	- 23288	135	9774.8 ± 149.3	−914.8	0.95	−738.8	0.95
	- 25863	121	10,076.3 ± 160.6	−370.2	0.95	−661.6	0.95
	- 27494	163	11,189 ± 150.1	+1636.8	0.96	+961.5	0.96
	- 28388	143	10,492.6 ± 170.2	+369.5	0.95	−724.9	0.95
	- AOM ± SE	18,545	10,287.1 ± 24.8	-	-	-	-
Rank-correlation value (r_rank_)				0.87 (*p* < 0.01)			

N * = number of progeny; BV = breeding value; b = reliability of the estimate; AFC = age at first calving; MY = 305-day milk yield the in first lactation; AOM = adjusted overall mean.

**Table 7:** Variance components and heritability estimates of the examined traits of Holstein-Friesian cows.

Parameters	AFC		MY	
	GLM Method	BLUP Animal Model	GLM Method	BLUP Animal Model
σ^2^_d_	1.17	0.71	963,784.4	819,426.0
σ^2^_e_	3.35	3.05	2,250,677.2	1,590,650.4
σ^2^_p =_ σ^2^_d +_ σ^2^_e_	4.52	3.76	3,214,461.7	2,410,076.4
h^2^ = σ^2^_d_/σ^2^_p_	0.26 ± 0.02	0.19 ± 0.01	0.30 ± 0.02	0.34 ± 0.01

AFC = age of first calving; MY = 305-day milk yield in the first lactation; σ^2^_d_ = genetic variance; σ^2^_e_ = residual variance; σ^2^_p_ = phenotypic variance; h^2^ = heritability.

**Table 8:** Phenotypic and genetic correlations between the examined traits of Holstein-Friesian cows.

r	Trait	MY
Phenotypic (r_p_)	AFC	−0.16 (*p* < 0.01)
Genetic (based on BV_GLM_ of sires) (r_g_)	AFC	−0.16 (*p* < 0.01)
Genetic (based on BV_BLUP_ of sires) (r_g_)	AFC	−0.09 (*p* < 0.05)
Genetic (based on BV_BLUP_ of entire population) (r_g_)	AFC	−0.05 (*p* < 0.01)

AFC = age of first calving; MY = 305-day milk yield in the first lactation; BV_GLM_ = breeding value estimated with the GLM method; BV_BLUP_ = breeding value estimated with the BLUP animal model.

**Table 9:** Trends in the examined traits of Holstein-Friesian cows.

Trend	Y	Slope (bX)			Intercept (a)			Fitting	
		b	SE	*p*	a	SE	*p*	R^2^	*p*
P	AFC	−0.12	0.02	<0.01	273.40	47.29	<0.01	0.75	<0.01
GSG	AFC^BV^	−0.05	0.01	<0.01	89.54	16.13	<0.01	0.69	<0.01
GSB	AFC^BV^	−0.01	0.01	NS	18.06	13.31	NS	0.12	NS
GAB	AFC^BV^	−0.01	0.00	<0.01	11.36	1.95	<0.01	0.42	<0.01
P	MY	+42.30	24.79	NS	−74,870.7	49,850.5	NS	0.25	NS
GSG	MY^BV^	+59.11	6.71	<0.01	−118,483.0	13,527.9	<0.01	0.81	<0.01
GSB	MY^BV^	+16.49	6.22	<0.05	−32,974.8	12,517.8	<0.05	0.29	<0.05
GAB	MY^BV^	+19.10	5.21	<0.01	−38,423.3	10,494.4	<0.01	0.60	<0.01

P = phenotypic trend; GSG = genetic trend, based on sire’s BV using the GLM method; GSB = genetic trend, based on sire’s BV using the BLUP animal model; GAB = genetic trend, based on all animals’ BV using the BLUP animal model; X = year of birth; AFC = average age at first calving (month); AFC^BV^ = average BV at AFC (month); MY = 305-day milk yield in the first lactation (kg); MY^BV^ = average BV at MY (kg).

## Data Availability

The data presented in this study are available on request from the National Association of Hungarian Holstein Friesian Breeders.
